# Binge-Like Exposure to Ethanol Enhances Morphine's Anti-nociception in B6 Mice

**DOI:** 10.3389/fpsyt.2018.00756

**Published:** 2019-01-22

**Authors:** Sulie L. Chang, Wenfei Huang, Haijun Han, Ilker K. Sariyer

**Affiliations:** ^1^Institute of NeuroImmune Pharmacology, South Orange, NJ, United States; ^2^Department of Biological Sciences, Seton Hall University, South Orange, NJ, United States; ^3^Department of Neuroscience, Temple University School of Medicine, Philadelphia, PA, United States

**Keywords:** morphine, mu opioid receptors, high-dose ethanol, anti-nociception, striatum, nucleus accumbens

## Abstract

Elevation of the blood ethanol concentration (BEC) to > 80 mg/dL (17.4 mM) after binge drinking enhances inflammation in brain and neuroimmune signaling pathways. Morphine abuse is frequently linked to excessive drinking. Morphine exerts its actions mainly via the seven transmembrane G-protein-coupled mu opioid receptors (MORs). Opioid use disorders (OUDs) include combination of opioids with alcohol, leading to opioid overdose-related deaths. We hypothesized that binge drinking potentiates onset and progression of OUD. Using C57BL/6J (B6) mice, we first characterized time-dependent inflammatory gene expression change as molecular markers using qRT-PCR within 24 h after binge-like exposure to high-dose, high-concentration ethanol (EtOH). The mice were given one injection of EtOH (5 g/kg, 42% v/v, i.g.) and sacrificed at 2.5 h, 5 h, 7.5 h, or 24 h later. Inflammatory cytokines interleukin (IL)-1β, IL-6, and IL-18 were elevated in both the striatum (STr) and the nucleus accumbens (NAc) of the mice. We then investigated the expression profile of MOR in the STr at 2 min, 5 h, or 24 h after the first EtOH injection and at 24 h and 48 h after the third injection. This binge-like exposure to EtOH upregulated MOR expression in the STr and NAc, an effect that could enhance morphine's anti-nociception. Therefore, we examined the impact of binge-like exposure to EtOH on morphine's anti-nociception at the behavioral level. The mice were treated with or without 3-d binge-like exposure to EtOH, and the anti-nociceptive changes were evaluated using the hot-plate test 24 h after the final (3rd) EtOH injection with or without a cumulative subcutaneous dose (0, 0.1, 0.3, 1.0, and 3.0 mg/kg) of morphine at intervals of 30 min. The response curve of the mice given EtOH was shifted to the left, showing enhanced latency to response to morphine up to 3 mg/kg. Furthermore, co-treatment with the MOR antagonist naltrexone blocked morphine's anti-nociception in animals given either EtOH or saline. This confirms that MOR is involved in binge-like exposure to EtOH-induced changes in morphine's anti-nociception. Our results suggest that EtOH enhanced latency to analgesic response to morphine, and such effect might initiate the onset and progression of OUDs.

## Introduction

Alcohol (EtOH) is the most widely used addictive substance in the world. The effects of alcohol drinking depend on the volume consumed, the concentration by volume, and the drinking pattern (1–4). Alcohol drinking patterns refers to different frequencies and amounts of alcohol intake, such as casual drinking, binge drinking, continuous drinking, frequent heavy drinking, and episodic drinking. National Institute on Alcohol Abuse and Alcoholism (NIAAA) has defined “binge drinking” as drinking enough EtOH in a short time to elevate the blood EtOH concentration (BEC) to > 80 mg/dL (5); that is, 17.4 mM. Binge drinking, particularly of hard liquor (> 40% alcohol by volume [ABV]), is a popular activity among adolescents (6). Hard liquor was involved in 43.8% of the binge drinking reported by subjects aged 13 to 20 yrs, with vodka being the most popular beverage (7). Epidemiologic studies indicate that adolescence is a risky period for initiation of EtOH use, and early onset is associated with a greater risk of late dependence or alcoholism (8–12). Alcohol consumption by adolescents also can lead to other addictive behaviors, including abuse of various other substances such as opioids, as well as neurocognitive deficits and social impairment. These pathological conditions may lead to direct and indirect changes in the neuromaturational course extending into adulthood (8–11). Not only chronic EtOH consumption, but also sporadic consumption, such as excessive weekend drinking, can provoke cognitive-deficit neuropsychological effects in young adults (13).

Binge drinking is observed in individuals with alcohol use disorders (AUDs). Chronic/repeated alcohol use alters nociception, including changes in pain sensation (14). Moreover, binge drinking induces gut leakage causing elevation of the blood endotoxin concentration (15, 16). This systemic endotoxin activity can trigger activation of inflammatory cytokines and has global effects on various cell types in different organs (17, 18). Numerous investigators have shown that binge drinking in humans and binge-like exposure to EtOH in animals encourages production of inflammatory molecules such as interleukin (IL)-1α, IL-6, IL-1β, and IL-18, as well as elevated activity of neuroimmune signaling pathways via various direct and indirect mechanisms (19, 20). Dysregulated continual synthesis of IL-6 has a pathological effect on chronic inflammation and autoimmunity (21). IL-1β is induced by pro-inflammatory signaling through Toll-like receptors (TLRs) or by cytokines, such as tumor necrosis factor (TNF)-α, IL-1β itself, and the inflammasome (22).

Morphine is a powerful, highly addictive opioid drug that exerts its analgesic action mainly via mu opioid receptors (MORs) (23). The MORs are also the principal site for morphine's induction of behavioral reward (24, 25), locomotion (26), analgesia (27), tolerance (28), and physical dependence (29). Naltrexone is a long-lasting competitive opioid antagonist that has high affinity for MORs (30, 31). Oral naltrexone has been used for many years to treat opioid dependence and has been approved since 1994 by the U.S. Food and Drug Administration to treat AUDs. Tail flick latency and hot-plate analgesia tests are common assays using rodent models to examine morphine's anti-nociception (32). MORs are involved in the interaction of morphine and EtOH, which induces neuroinflammation (33, 34).

We have reported that treatment with the pro-inflammatory cytokine IL-1β significantly increases MOR expression in endothelial cells (35) and in human U87 MG cells (36). In another *in vitro* study, we reported that the upregulation of MOR induced by lipopolysaccharide (LPS) stimulation in macrophage-like TPA-HL-60 cells and conditioned medium from LPS-stimulated TPA-HL-60 cells increases MOR expression in SH-SY5Y cells, a neuronal cell model, through actions mediated by TNF-α and granulocyte–macrophage colony-stimulating factor (37). The LPS-challenged HIV-1 transgenic (HIV-1Tg) rat model with neuroinflammation demonstrates an increase in MOR expression and is more sensitive to morphine's effect in the conditioned place preference test (38, 39).

Taken together, considering the above-mentioned studies showing that (1) binge-like exposure to EtOH induces inflammation and inflammatory cytokines (19, 20) and (2) inflammatory cytokines mediate expression of MOR (35–37) and change morphine actions (38, 39), we hypothesized that binge-like exposure to EtOH increases expression of MOR and changes morphine-induced anti-nociception by inducing elevation of inflammatory molecules in the brain. In the present study, adolescent C57BL/6J mice were given binge-like exposure to high-dose, high-concentration EtOH for 3d by intragastric (i.g.) injection to mimic underage binge alcohol drinking, such as over a weekend (40). The blood EtOH concentration (BEC) after single and repeated EtOH administration was measured. Time-dependent gene expression change was investigated using qRT-PCR as molecular markers to evaluate the response to this binge-like exposure to EtOH. The nucleus accumbens (NAc) plays an important role in processing rewarding and reinforcing stimuli including drug addiction; the striatum (STr) is part of the brain's reward circuit and a key region responsible for voluntary motor control (41). Therefore, we studied expression of the pro-inflammatory cytokine genes *Il1b, Il6*, and *Il18*, as well as the MOR gene *Oprm1*, in the NAc and STr. Finally, hot-plate tests were employed to evaluate the behavioral effect of binge-like exposure to EtOH on morphine's anti-nociception. The opioid antagonist naltrexone was used to confirm morphine's action on MOR. Our results suggest that neuroinflammation induced by binge-like exposure to EtOH contributes to elevation of morphine's anti-nociception response. Such a change might be one of the fundamental mechanisms underlying encouragement of OUDs by binge-like EtOH exposure.

## Materials and Methods

### Animals

C57BL/6J mice (3–4 wks old) were purchased from the Jackson Laboratory (Bar Harbor, ME). They were housed with four animals per ventilated plastic cage (Animal Care Systems Inc., Centennial, CO) and maintained in a temperature- and humidity-controlled environment. They were kept on a 12-h light/dark cycle and fed a standard rodent diet. The experimental protocol was approved by the Institutional Animal Care and Use Committee (IACUC) at Seton Hall University, South Orange, NJ.

### EtOH Treatment and BEC Determination

The mice were allowed at least one week to adapt to the facility. To minimize the non-specific stress response to i.g. injection of EtOH, the adolescent mice (at ~ 5 wks) were given 2-day conditioning by intragastric (i.g.) injection of water. The first group of mice was then given one dose of 5 g/kg/d of 42% v/v EtOH as a bolus via i.g. injection. Tail vein blood was collected by tail clipping prior to and at 10 min, 20 min, 1 h, 2 h, 4 h, 6 h, and 8 h after treatment. A second group of mice was designated to receive the same dose of EtOH for 1, 2, or 3 d; and blood was collected 5 h after the last injection (Table 1). Plasma was obtained by centrifugation of whole blood at 10,000 rpm for 10 min at 4°C and stored at −80°C until analysis. The EtOH concentration was determined using an Ethanol Assay Kit (Biovision, Milpitas, CA) following the manufacturer's instructions. The BEC data were analyzed using Student's *t*-test.

**Table 1:** EtOH administration and determination of blood EtOH concentration.

**Treatment**	**Day 1**	**Day 2**	**Day 3**
Water			Blood collection
EtOH 2 min			Blood collection 2 min after injection
EtOH 1 d 5 h			Blood collection 5 h after injection
Repeated EtOH 2 d 5 h		EtOH	Blood collection 5 h after injection
Repeated EtOH 3 d 5 h	EtOH	EtOH	Blood collection 5 h after injection

*As vehicle control, water injections (not shown) were given*.

### EtOH Treatment and Tissue Collection

After 2-day conditioning, the B6 mice were designated to receive 5 g/kg 42% v/v EtOH as a bolus one time and sacrificed at 2 min, 2.5 h, 5 h, 7.5 h, or 24 h after treatment, after which the brains were microdissected. The STr and NAc were stored at −80°C until analysis. A second batch of B6 mice received the same dose of EtOH for 1 or 3 days (Table 2). These mice were sacrificed 5 h after the last injection. By adapting the EtOH treatment regimen as reported previously (42), we conducted preliminary studies using animals receiving water or EtOH for 2 min. Other than the BEC reading, there are no significant differences between the readings of various assessments on the animals sacrificed immediately (2 min) after receiving EtOH (EtOH for 2 min) and those of the animals receiving water. For example, in the STr, ΔCt of *Il1b* was 8.77 ± 0.22 in the water group and 8.51±0.58 in the EtOH for 2 min group, with a fold change of 1.20 ± 0.38 (*p* = 0.38); and in the NAc, ΔCt of *Il1b* was 9.11 ± 0.52 in the water group and 9.42±0.53 in the EtOH for 2 min group, with a fold change of 1.74 ± 0.91 (*p* = 0.10). The above data were reproduced in two additional experiments. For the time course study of gene expression changes, it is necessary to include 2-min EtOH group that was used as control for data analysis. In line with IACUC and NIH guideline to minimize use of the animals, no water group was included in the study for Oprm1 daily expression following binge-like exposure to EtOH. As reported previously (42), we have used 2-min EtOH as control throughout this research project. Brains were microdissected, and the STr and NAc were collected and stored at −80°C until use.

**Table 2:** EtOH administration timeline for *Oprm1* response.

**Treatment**	**Day 1**	**Day 2**	**Day 3**	**Day 4**	**Day 5**
EtOH 2 min				EtOH; sacrifice 2 min after injection	
EtOH 5 h				EtOH; sacrifice 5 h after injection	
EtOH 24 h			EtOH	Sacrifice	
Repeated EtOH 24 h	EtOH	EtOH	EtOH	Sacrifice	
Repeated EtOH 48 h	EtOH	EtOH	EtOH		Sacrifice

*As vehicle control, water injections (not shown) were given*.

### RNA Isolation and cDNA Preparation

Total RNA was extracted from the STr and NAc using the RNeasy Mini Kit (Qiagen, Germantown, MD), followed by RNase-free DNase (Qiagen) digestion to remove contaminating DNA. The RNA quality and quantity were determined using an ND1000 Nanodrop spectrophotometer (Thermo Scientific, Waltham, MA) and verified by gel electrophoresis. An equal amount of RNA (400 ng) from each sample was converted to cDNA using the RT^2^ First-Strand Kit (Qiagen) according to the manufacturer's instructions.

### qRT-PCR Analysis

Gene expression was quantified using RT^2^ SYBR ROX qPCR Master Mix (Qiagen) as described previously (38, 40, 43). Real-time polymerase chain reaction (PCR) was performed with the ABI Prism 7900HT Fast Detection System (Applied Biosystems, Foster, CA). The thermocycler parameters were 95°C for 10 min followed by 40 cycles at 95°C for 15 s and 60°C for 1 min. ROX was used as the passive reference. Expressions of all genes were normalized to expression of β-actin (*Actb*) and splicing factor, arginine/serine-rich 4 (*Sfrs4*). The relative expression of each gene was compared with expression of that gene in the mice given EtOH for 2 min and calculated using the ΔΔCT method (44). The primer sequences for *Il1b, Il6, Oprm1, Actb*, and *Sfrs4* are listed in Table 3. The *Il18* primers were purchased from Qiagen (Cat No. PPM03112B). Data were analyzed using one-way ANOVA followed by Dunnett's post-tests in GraphPad Prism 5 software (GraphPad Software Inc., La Jolla, CA).

**Table 3:** PCR array primer sequences.

**Gene symbol**	**Primer**	**Sequence 5^**′**^3^**′**^**
*Oprm1*	Forward	CCAGGGAACATCAGCGACTG
	Reverse	GTTGCCATCAACGTGGGAC
*Il1b*	Forward	AATGCCACCTTTTGACAGTGATG
	Reverse	GGAAGGTCCACGGGAAAGAC
*Il6*	Forward	CCCCAATTTCCAATGCTCTCC
	Reverse	GGATGGTCTTGGTCCTTAGCC
*Actb*	Forward	GGCACCACACCTTCTACAATG
	Reverse	GGGGTGTTGAAGGTCTCAAAC
*Sfrs4*	Forward	GATCTGAAGAACGGGTATGGCT
	Reverse	ACACAGGTCTTTGCCGTTCA

### EtOH Treatment and Hot-Plate Tests

Male 5-week-old B6 mice were designated to receive either 5 g/kg 42% v/v EtOH or water (control) daily for 3 days. Morphine sulfate (Sigma, St. Louis, MO) was freshly prepared prior to use by dissolving it in 0.9% sterile saline. A 1.0-mg/mL morphine solution was serially diluted to create doses of 0.1, 0.3, 1.0, or 3.0 mg/kg. A saline solution with no morphine was the control for morphine treatment. In our preliminary studies, the animals were given cumulative doses of morphine of 0.1, 0.3, 1.0, 3.0, and 10 mg/kg to select the morphine doses to be used. On subcutaneously (s.c.) treatment with morphine at 10 mg/kg, both control and experimental animals presented abnormal behaviors that were beyond measurement using the hot-plate test. Therefore, we chose morphine doses of 0.1, 0.3, 1.0, and 3.0 mg/kg.

As shown in Table 4, on the day after Day 3 of binge-like exposure to high-dose, high-concentration EtOH, the mice were injected subcutaneously (s.c.) with a cumulative dose of morphine as noted above at intervals of 30 min and placed on the hot plate of the IITC Test Analgesia Meter (Woodland Hills, CA) that was set at 55°C. The latency was recorded according to hind-paw lick or jumping on the meter. A maximum 120-s cutoff was set to avoid tissue damage. The latency (s) was plotted against morphine doses (45). By adhering to the IACUC and NIH guideline, the minimum number of animals needed to obtain statistical power was discovered and used.

**Table 4:** EtOH administration and hot-plate test timeline.

**Treatment**	**Day 1—Day 3**	**Day 4**
Water alone	Daily water injections	Hot-plate tests
Water + morphine	Daily water injections	Cumulative doses of morphine (s.c.) and hot-plate tests
EtOH alone	Daily EtOH injections	Hot-plate tests
EtOH + morphine	Daily EtOH injections	Cumulative doses of morphine (s.c.) and hot-plate tests

In a parallel experiment, naltrexone (1 mg/kg) was administrated s.c. 5 min prior to morphine injection. Hot-plate test results were analyzed using two-way repeated measures ANOVA, followed by Bonferroni post-tests in GraphPad Prism 5 software (GraphPad Software Inc., La Jolla, CA) (46).

## Results

### Time Course of Blood EtOH Concentration of Mice Given Single Binge-Like Exposure to High-Dose, High-Concentration EtOH

**Figure 1:**
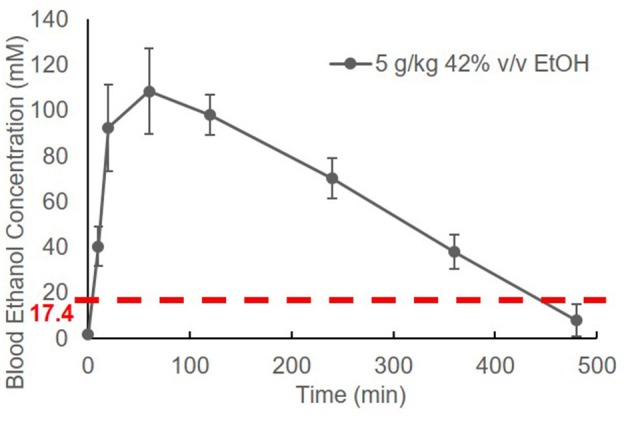
Time course of blood EtOH concentration. Three B6 mice were given EtOH (5 g/kg, 42% v/v, i.g.). The BEC was measured prior to (0 min) and at 10 min, 20 min, 1 h, 2 h, 4 h, 6 h, and 8 h later and normalized by subtracting background (0 min BEC) and plotted against time.

The BEC of adolescent C57BL/6J (B6) mice was measured prior to and at 10 min, 20 min, 1 h, 2 h, 4 h, 6 h, and 8 h after EtOH administration. Striking elevation of the BEC to approximately 100 mM was observed at 20 min, and it reached a peak of 108.4 ± 18.8 mM at 1 h. The BEC then declined gradually. After a single binge-like exposure, the time required for the animal's BEC to reach < 17.4 mM was close to 8 h (Figure 1).

### Blood EtOH Concentration of Mice Given Repeated Binge-Like Exposure to High-Dose, High-Concentration EtOH

**Figure 2:**
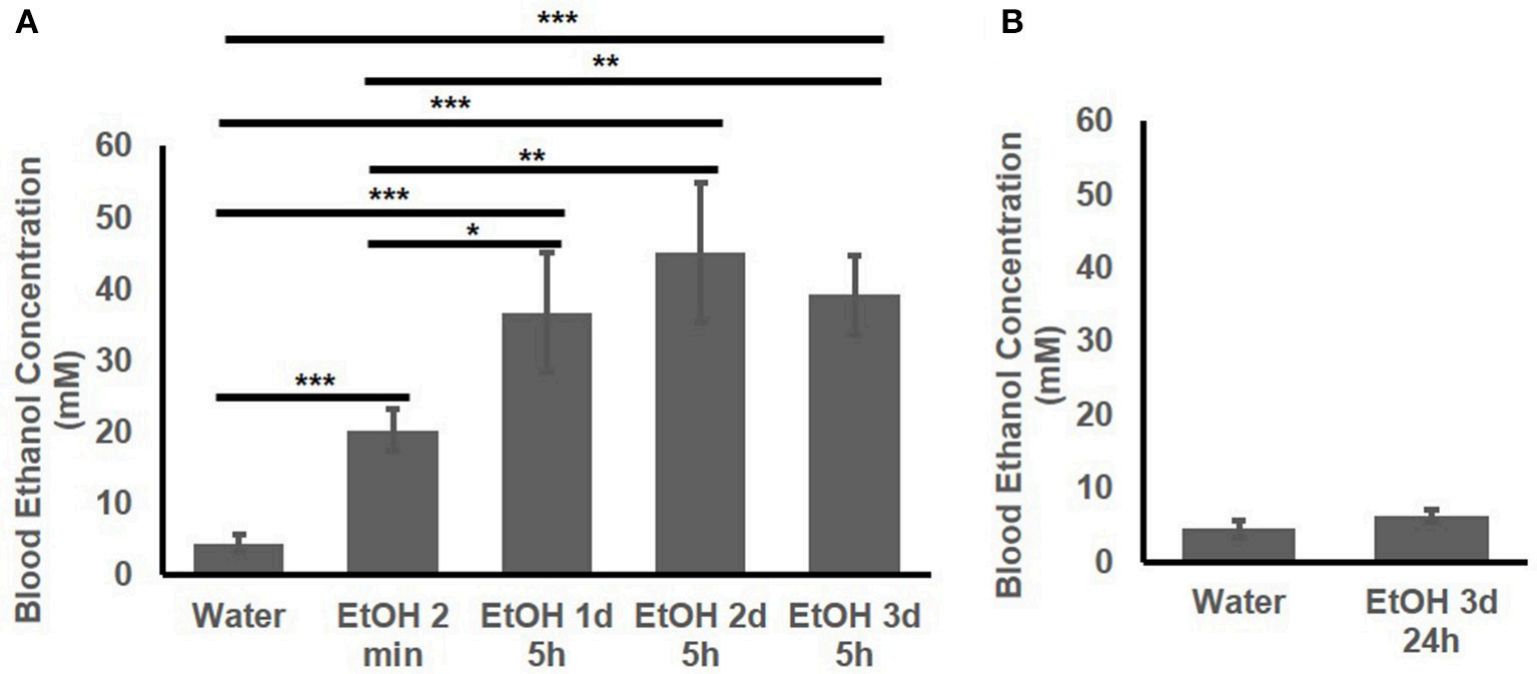
Blood EtOH concentration after repeated EtOH administration (5 g/kg/d; 42% v/v; i.g.)**. (A)** Concentrations at 5 h after 1st, 2nd, and 3rd administration. **(B)** 24 h after 3 d of administration, BEC was back to basal concentration. Statistical analysis was performed using student's t tests. ^*^p < 0.05, ^**^p < 0.01, ^***^p < 0.001; *n* = 4.

The BEC of the adolescent B6 mice treated with 1 d, 2d, or 3 d of high-dose. high-concentration EtOH was determined. At 2 min after EtOH treatment, the BEC had already risen to 20.25 ± 2.89 mM. The BEC at 5 h after the 1st, 2nd, and 3rd EtOH injection were compared with that of the mice given water and at 2 min after EtOH injection (Figure 2A). At 5 h after EtOH administration, the BEC was significantly higher than at 2 min and the basal BEC of the mice given water. There was no significant difference in the BEC at 5 h after repeated EtOH administration on different days.

Figure 2B shows that at 24 h after the 3rd EtOH delivery, the BEC had returned to the basal concentration. There was no significant difference in the BEC of these mice compared with that of the mice given water.

### Elevated Inflammatory Molecule Expression After Single Binge-Like Exposure to High-Dose, High-Concentration EtOH

**Figure 3:**
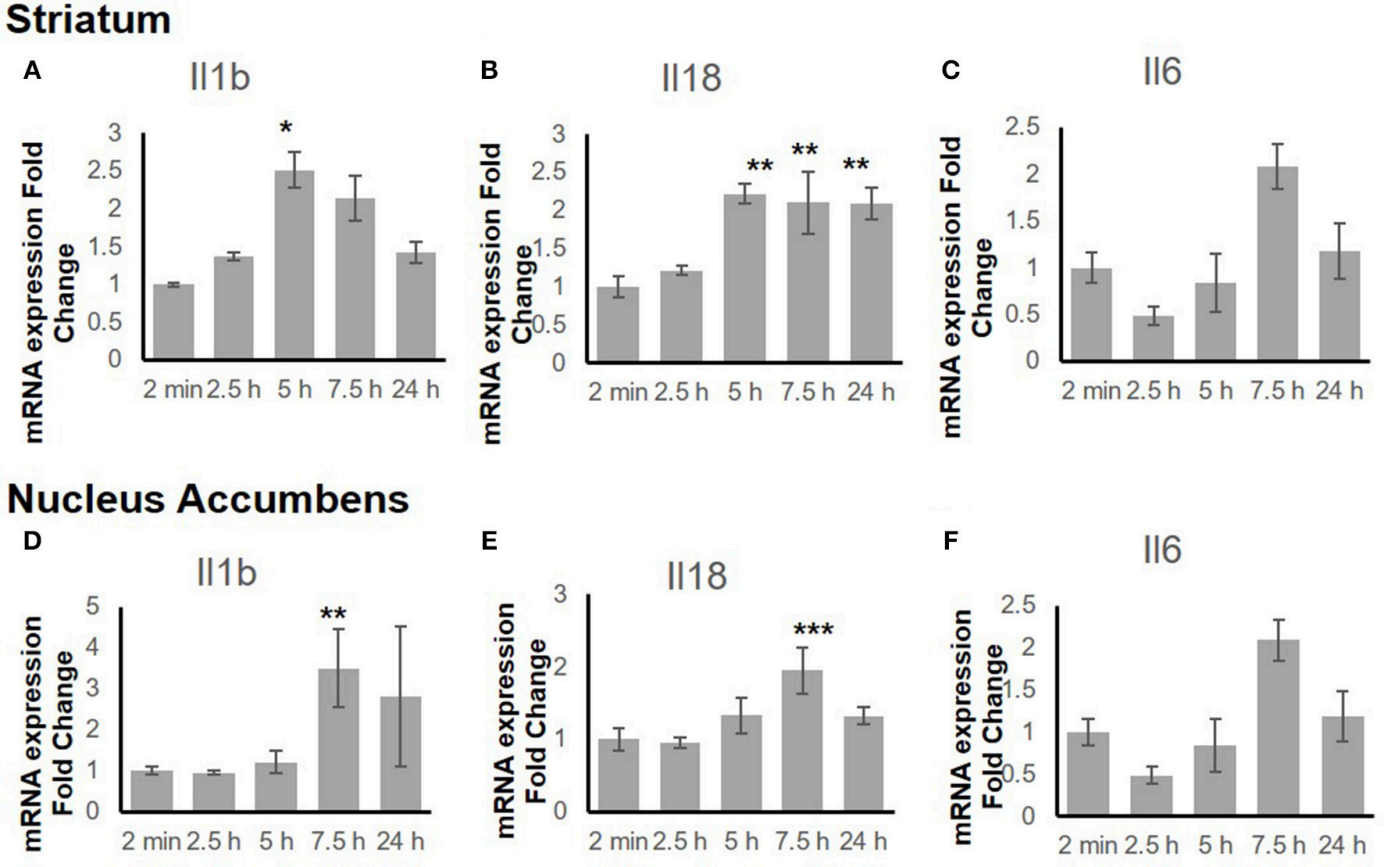
Inflammation-related gene expression change in striatum **(A–C)** and nucleus accumbens **(D–F)** within 24 h in response to binge-like EtOH administration (42% v/v, 5 g/kg, i.g.). Data are expressed as mean ± SE. Statistical analysis was performed using one-way ANOVA followed by Dunnett's post-tests, compared with control 2-min EtOH group: ^*^p < 0.05, ^**^p < 0.01, ^***^p < 0.001; *n* = 4.

To explore the time-dependent response of the inflammatory genes, the gene expression change in both the STr and the NAc at 2 min, 2.5 h, 5 h, 7.5 h, and 24 h after binge-like exposure to EtOH was determined. The qRT-PCR revealed that proinflammatory genes *Il18* (F_(4, 15)_ = 9.66, *p* < 0.001) and *Il1b* [*F*_(4, 14)_ = 3.21, *p* < 0.05] showed significant changes in the STr within 24 h following EtOH exposure (Figures 3A,B). Expression of *Il1b* (*p* < 0.05) and *Il18* (*p* < 0.01) increased significantly at 5 h after EtOH treatment; *Il18* expression remained high until 24 h (*p* < 0.01). Within the time course of 24 h, anti-inflammatory *Il6* showed a significant change in STr [*F*_(4, 14)_ = 3.26, *p* < 0.05]; *Il6* increased at 7.5 h but fell after 24 h in STr (Figure 3C).

Figures 3D–F shows a late response in expression of inflammatory genes *Il18* [*F*_(4, 14)_ = 11.10, *p* < 0.001] and *Il1b* [*F*_(4, 14)_ = 5.85, *p* < 0.01] in the NAc. At 7.5 h after binge-like exposure to high-dose, high-concentration EtOH, pro-inflammatory *Il1b* (*p* < 0.01) and *Il18* expression (*p* < 0.001) was significantly elevated at 7.5 h. Meanwhile, the extent of anti-inflammatory *Il6* decreased [*F*_(4, 14)_ = 0.81, *p* > 0.05]. After 24 h, the extent of *Il1b, Il18, and Il6* expression did not show a significant difference from that in the 2-min control group.

### Repeated Binge-Like Exposure to High-Dose, High-Concentration EtOH Induced Upregulation of MOR Expression

**Figure 4:**
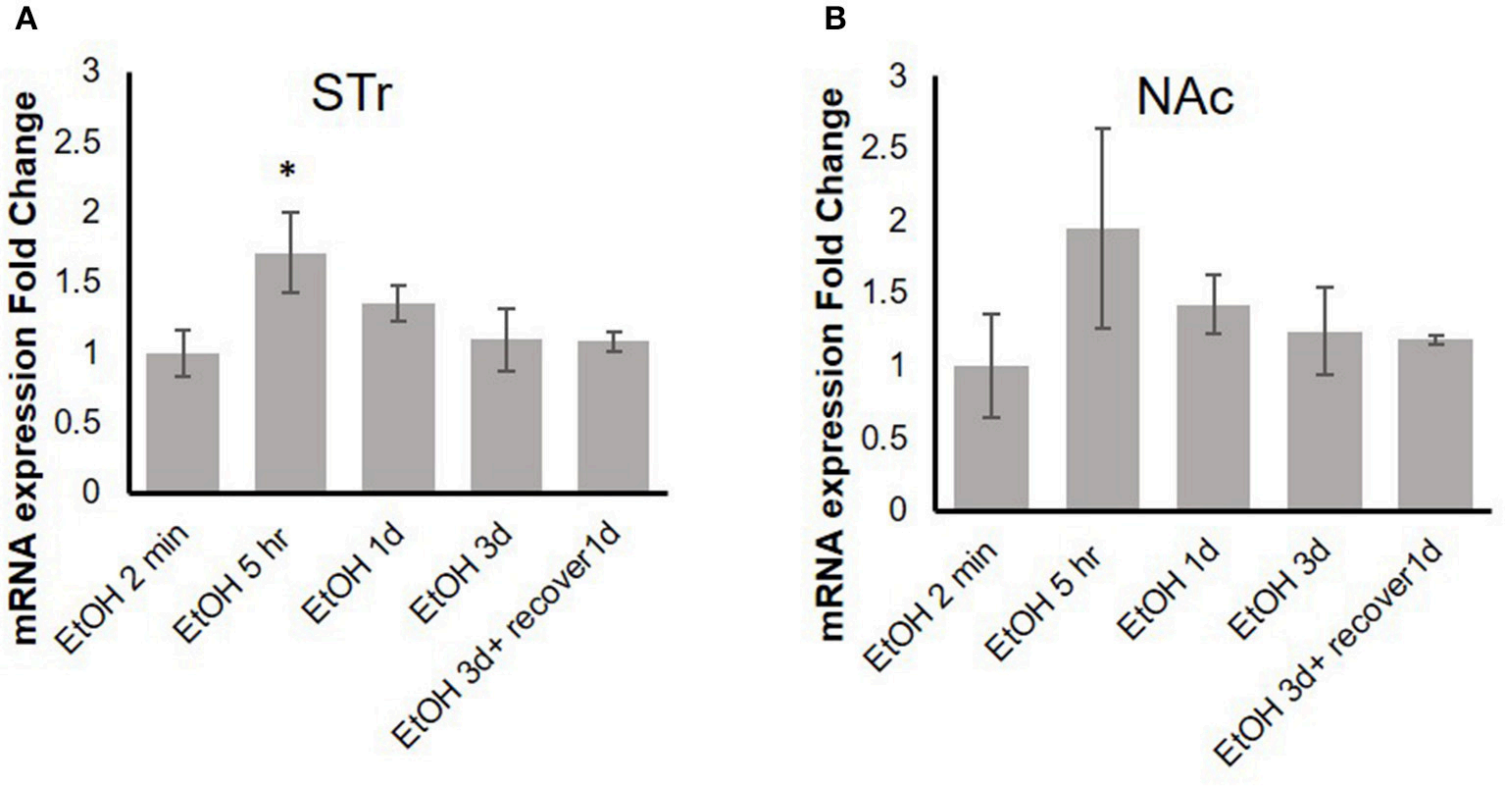
Time-dependent expression change of MOR gene, *Oprm1*, in striatum **(A)** and nucleus accumbens **(B)** in response to binge-like EtOH administration (42% v/v, 5 g/kg/d, i.g.). Data are presented as mean ± SD. Statistical analysis was performed using one-way ANOVA followed by Dunnett's post-tests compared with control 2-min EtOH group: ^*^p < 0.05; *n* = 4.

Our previous *in vitro* studies showed that MOR expression is induced by pro-inflammatory cytokines (36, 37), and therefore, we examined the time course of mRNA expression of the MOR gene *Oprm1* in the STr and NAc of brains of binge-like EtOH-treated B6 mice at 2 min, 5 h, or 24 h after the first EtOH infusion and at 24 and 48 h after the third infusion. In the STr, expression of *Oprm1* had increased significantly by 5 h after the first EtOH delivery and then gradually declined [*F*_(4, 9)_ = 4.25, *p* < 0.05]; at 5 h after EtOH injection, *Oprm1* expression was significantly higher than that at 2 min (*p* < 0.05) (Figure 4A). Figure 4B shows a similar trend for *Oprm1* in the NAc [*F*_(4, 9)_ = 1.95, *p* > 0.05] (Figure 4B).

### Binge-Like Exposure to High-Dose, High-Concentration EtOH Alters Morphine's Anti-nociception

**Figure 5:**
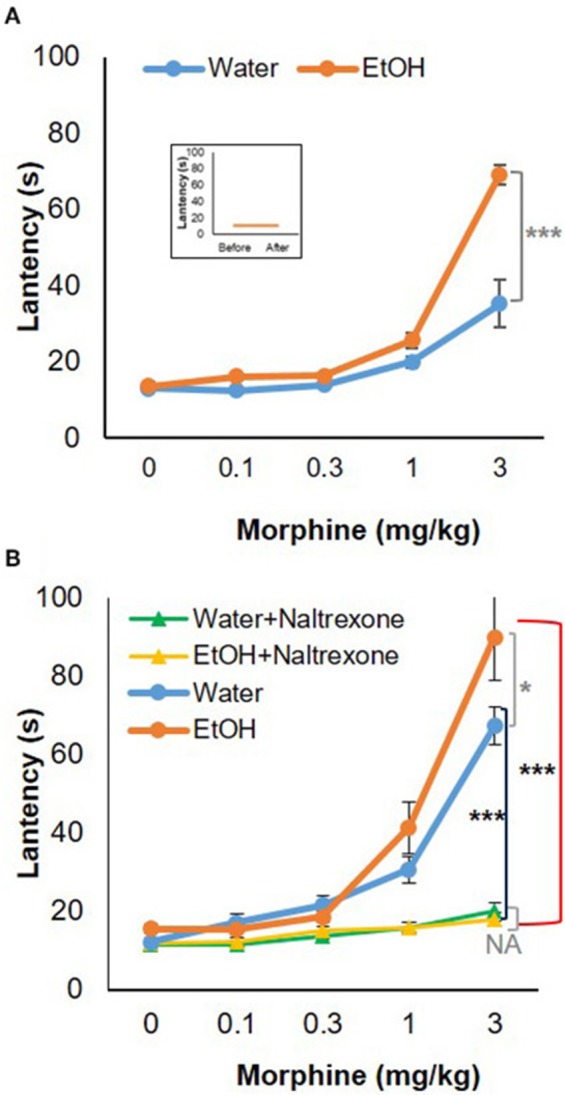
Hot-plate analgesia test in response to morphine in 5-week-old B6 mice given 3-d binge-like EtOH treatment. **(A)** Ethanol-treated mice (*n* = 4) showed elevated response to morphine; morphine response curve was shifted to the left. (Insert) the animals given both water and EtOH didn't show change in latency without morphine injections (*n* = 4). **(B)** The response latency shift was completely ablated by opioid receptor antagonist naltrexone (1 mg/kg; s.c.) (*n* = 3). Statistical analysis was performed using two-way repeated measures ANOVA followed by Bonferroni post-tests. ^*^p < 0.05, ^***^p < 0.001.

To test the behavioral consequences of the gene expression change induced by binge-like exposure to high-dose, high-concentration EtOH, hot-plate tests were performed to evaluate morphine's anti-nociception effect. 24 h after the 3rd d EtOH injection, the mice were injected s.c. with a cumulative dose (0, 0.1, 0.3, 1.0, or 3.0 mg/kg) of morphine at intervals of 30 min and then placed on a 55°C hot plate. As shown in the insert in Figure 5A, hot plate latency of the mice given either water or EtOH alone didn't change with no morphine injections. Of the mice given water, the latencies were 10.68 ± 3.52 s and 10.89 ± 2.79 s prior to and after injections, respectively; of the animals given EtOH, the latency readings were 10.93 ± 3.22 s and 10.88 ± 4.04 s, respectively. Morphine produced dose-dependent anti-nociception both in animals given water and in those receiving EtOH. In comparison with the animals given water (blue curve), the animals receiving EtOH showed a greater response to morphine; the response curve was shifted to the left [*F*_(4, 120)_ = 5.73, *p* < 0.001] (Figure 5A). The latency to analgesic response was significantly enhanced in EtOH-treated animals at 3 mg/kg dose of morphine (*p* < 0.001). The response latency induced by morphine was ablated by naltrexone in animals treated with EtOH [*F*_(4, 72)_ = 42.78, *p* < 0.001] or water [*F*_(4, 88)_ = 13.20, *p* < 0.001], and no difference was observed between animals given EtOH and those with water [*F*_(4, 55)_ = 0.87, *p* > 0.05] (Figure 5B).

## Discussion

“Binge drinking” is repeated EtOH intake causing a BEC > 80 mg/dL (17.4 mM) (5). The peak BEC of binge drinkers, from 18 to 50 years old or older, has been reported to be as high as 470 mg/dL (that is equal to 102 mM) (47). In addition to the well-characterized liver toxicity, binge drinking can cause various neurologic disorders (48). Morphine use/abuse frequently is linked to drinking, especially excessive drinking. Combining opioids with other substances, including EtOH, increases opioid overdose deaths (49). During the last decade, an intertwined epidemic of drug abuse and addiction, EtOH addiction, and binge drinking has emerged (50).

Alcohol research investigators have commonly used rodent models to mimic human alcohol consumption, particularly specific drinking patterns such as binge drinking (40, 51, 52). The high dose of EtOH we chose to administer (5 g/kg; 42% v/v) is equivalent to the alcohol by volume (ABV) of the hard liquors, such as vodka. By adapting the high dose of EtOH used in many rodent studies, the specified dose of EtOH was used in our study. The dose of 5 g/kg is well-established as a binge-drinking model in mice (40, 52). To differentiate binge drinking in humans from the model in mice, we have used the term “binge-like exposure to EtOH.” Figure 1 shows that a BEC higher than 17.4 mM was detected in mice after one treatment (i.g.) with EtOH (5 g/kg). The elevation of BEC above 17.4 mM was detected within 2 min (Figures 1, 2A), and the reading reached its peak, 108.4 ± 18.8 mM, at 1 h. The peak declined gradually over 7–8 h. The instant rise of BEC to the NIAAA-defined binge concentration (17.4 mM) and the prolonged high concentration of alcohol could exert significant systemic effects, including intoxication, overburden of the liver for alcohol metabolism, and early and transient pro-inflammatory states (53–56).

Underage drinking, including binge drinking over the weekend, is common (7). To mimic the underage common drinking pattern, we chose 3-d high-dose, high-concentration EtOH dose. After each of the three binge-like EtOH treatments, the instant elevation of the BEC to > 17.4 mM and the long duration of the elevated BEC followed by a reduction to below 17.4 mM on Day 1 were also observed on Day 2 and Day 3 (Figure 2A). At the 5 h point, the BEC was 35–40 mM on all 3 days (Figure 2A). The 3-d binge-like exposure to EtOH therefore gave the animals the BEC > 17.4 mM for ~ 24 h in total. We previously reported that this 3-d high-dose, high-concentration EtOH binge-like regimen induces a stress response in the hippocampus of adolescent rats, and the downstream effects of the EtOH-induced stress response in the hippocampus appear to be involved in reduction of the spleen size (40).

In addition, there was a significantly higher plasma endotoxin concentration (200 EU/mL) in the animals given 3-d binge-like exposure to EtOH (40). Other studies have found that binge drinking in human subjects, as well as binge-like exposure to EtOH in rodents, induces gut leakage that elevates the blood endotoxin concentration (15, 16), which leads to production of inflammatory molecules, as well as greater activity of neuroimmune signaling pathways (19, 20). As noted previously, binge-like exposure to high-dose, high-concentration EtOH can trigger a severe immune response that persists even after EtOH has been metabolized (Figures 1, 2) (57).

After administration of EtOH, this volatile compound distributes into the cytosol of all cells. Thus, in addition to the hippocampus in which the 3-d binge-like exposure to EtOH induces stress responses (40), this EtOH regimen is expected to affect other brain areas, including those responsible for changes in pain sensation, as it was previously reported that binge EtOH consumption increases inflammatory pain responses and mechanical and cold sensitivity (14). We focused on the STr and the NAc areas. The STr is part of the brain's reward circuit and a key region responsible for voluntary motor control (41, 58). The STr projects to the basal ganglia, a neuronal circuit necessary for voluntary movement control, and exerts neuronal activity related to movement, rewards, and the conjunction of movement and reward (41, 59). The MOR is highly expressed in the STr (60, 61). The NAc plays an important role in the generation of motivated behaviors (62) and facilitates reward seeking by integrating neurotransmitter-mediated reinforcement signals with environmental stimuli (63, 64).

Figure 3 shows the mRNA time courses of the expression of *Il1b, Il18, and Il6* genes in both the STr and the NAc after one binge-like exposure to high-dose, high-concentration EtOH. The initial elevation of the expression these three genes in the STr appeared between 5 and 7.5 h, whereas only *Il18* remained significantly elevated at 24 h. However, in the NAc, the significant elevation of the products of these 3 genes was detected only at 7.5 h, and the elevation did not last to 24 h. The elevation time point and differential duration appeared to be brain-region dependent and suggest that EtOH-mediated effects are more intense in the STr than in the NAc because the STr projects to the NAc (65, 66).

Previous studies have demonstrated that expression of *Oprm1* is stimulated by various pro-inflammatory cytokines, including IL-1β (35–37). Figure 4 shows that in the STr, significant upregulation of *Oprm1* was observed at 5 h after binge-like exposure to EtOH. On the other hand, there was only a trend to elevation of *Oprm1* in the NAc. Although *Oprm1* mRNA elevation disappeared by the 24-h time point, protein level of the mu opioid receptors might stay elevated; confirmation of this idea is needed. Following binge-like exposure to EtOH, inflammatory cytokines and *Oprm1* mRNA levels both change in the brain areas. However, the concurrent elevation of these genes might imply, but not confirm, the direct correlation between expression of inflammatory cytokines and *Oprm1* induced by high-dose, high-concentration EtOH. Our qPCR data showed that binge-like exposure to EtOH caused both neuroinflammation and upregulation of MOR in various brain areas. Further studies are on the way to examine the causal correlation between expression elevation of inflammatory cytokines and *Oprm1* following binge-like exposure to EtOH.

Elevation of *Oprm1* implies increased expression and activity of MOR. Anti-nociception associated with morphine use would be the behavioral outcome of this elevation. Figure 5A shows morphine anti-nociception in adolescent C57BL/6J mice as determined by hot-plate analgesia tests at 24 h after 3-d binge-like EtOH treatment. There was an increase in morphine-induced anti-nociception after the EtOH treatment (Figure 5). Co-treatment with naltrexone, the selective MOR antagonist, abolished anti-nociception of the cumulative dosage of morphine in the mice given either binge-like exposure to EtOH or saline. This suggests that MOR is involved in morphine's anti-nociception elevation by binge-like exposure to EtOH. This also confirms that the 3-d EtOH at a high dose (5 g/kg) and high concentration (42% v/v) contributed to elevation of neuroinflammation and expression of MOR.

As noted previously, morphine abuse is frequently linked to excessive drinking. A cross-tolerance could take place between EtOH intake and treatment with morphine that is the high-affinity agonist for MOR. Le et al reported that in adult male rats, chronic EtOH consumption decreases the response to treatment with morphine (67). He et al reported that repeated EtOH intake by self-administration (5–6 g/kg/24 h) decreases the anti-nociception of MOR agonists. Inhibition of MOR endocytosis is a possible mechanism underlying the cross-tolerance interaction between EtOH and MOR agonists (68). Shah et al reported that chronic EtOH consumption, but not a single injection that resulted in a BEC of approximately 15 mg/dL, decreases the analgesic potency of opioids in mice. However, the investigators were not sure of the mechanism underlying the interaction between EtOH and opioids, including morphine (69). In examining the alleviation of CRF1 receptor antagonism related to heroin and EtOH dependence, Edwards et al suggested that understanding the relations between chronic exposure to addictive substances such as EtOH and pain-related states such as nociception could reveal the mechanisms underlying the transition to addiction to various substances of abuse (70). Other than the study reported by Shah et al, all these studies suggested how treatment with EtOH changed the activity of MOR and MOR-mediated morphine-induced anti-nociception. Taking these data together with the studies showing that inflammatory cytokines mediate expression of MOR (35–37) and change morphine actions (38, 39), we have reconciled two of our previous studies in light of our current study to address how inflammation induced by various exogenous challenges such as binge drinking might change the subject's response to morphine's anti-nociception.

In one of our previous studies, we used HIV-1 transgenic (HIV-1Tg) rats, mimicking people living with HIV/AIDS and receiving combination antiretroviral therapy (cART), to demonstrate that the persistent presence of HIV-1 proteins elevates inflammation in the brain that possibly correlates with upregulation of MOR expression and the enhancement of morphine's anti-nociception (71, 72). In another study, using F344 rats, we showed that repeated treatment with LPS elevates inflammation in the brain and enhances the sensitivity to morphine's anti-nociception and morphine-induced conditioned place preference (73). With binge-like exposure to high-dose, high-concentration EtOH in adolescent mice, with the persistent presence of HIV proteins in the HIV-1Tg rats (71, 72), and with repeated treatment with LPS there was enhancement of morphine's anti-nociception secondary to upregulation of MOR expression that might be the outcome of elevation of inflammation in the brain. Taken together, our three studies appear to confirm that systemic inflammation attributable to the persistence of viral proteins, repeated treatment with LPS, or binge-like exposure to EtOH leading to elevation of plasma endotoxin, enhanced the rewarding effects of morphine, both physiologically and behaviorally, thereby increasing the potential for morphine abuse and addiction.

In summary, our research indicated that binge-like exposure to high-dose, high-concentration EtOH- enhanced morphine anti-nociception might be mediated via elevation of neuroinflammation. Because morphine is highly addictive, alteration of the animals' response to its use in the course of systemic inflammation could cause the onset and progression of OUDs in the course of inflammation following binge-like exposure to EtOH. As a result of the current study, mega-analysis using bioinformatics tools to link neuroinflammation parameters, expression of MOR, and determinants of nociception will be conducted to extend the findings of our current study.

## Author Contributions

SC designed the studies; participated in data collection, data analysis, and manuscript preparation; and approved the manuscript submission. WH conducted the animal treatments and PCR array analysis and participated in tissue collection and manuscript preparation. HH conducted the hot-plate tests and participated in tissue collection. IS participated in data interpretation and manuscript preparation.

### Conflict of Interest Statement

The authors declare that the research was conducted in the absence of any commercial or financial relationships that could be construed as a potential conflict of interest.
